# On the Role of Color in Reading and Comprehension Tasks in Dyslexic Children and Adults

**DOI:** 10.1177/2041669518779098

**Published:** 2018-06-09

**Authors:** Baingio Pinna, Katia Deiana

**Affiliations:** Department of Biomedical Sciences, University of Sassari, Italy

**Keywords:** color vision, developmental dyslexia, perceptual organization, reading, biology of color vision

## Abstract

We investigated the effect of chromatic variations on the reading process with normal and dyslexic readers. We demonstrate that color can induce wholeness, parts-whole organization and phenomenal fragmentation during reading and comprehension tasks within written texts made up of words and non-words in the following conditions: monochromatic (the whole text colored with only one color), word (each word colored in different color), half word (half word colored with a color different from the one of the second half), syllable (every syllable colored with a different color) and letter (each letter with a different color). The dependent variables considered were reading time, reading errors and incorrect answers to a comprehension test. The main results demonstrated that these parameters of reading performance are all influenced by the five aforementioned chromatic conditions. These outcomes manifest similar trends in four groups of readers: children and adults combined with normal or dyslexic readers. Possible applied research and clinical applications are discussed together with some basic questions related to color vision suggesting that the main purposes of color for living beings is to generate wholeness, parts-whole organization and perceptual segmentation.

## Introduction

### Biological and Visual Meanings of Color

**Figure 1.**
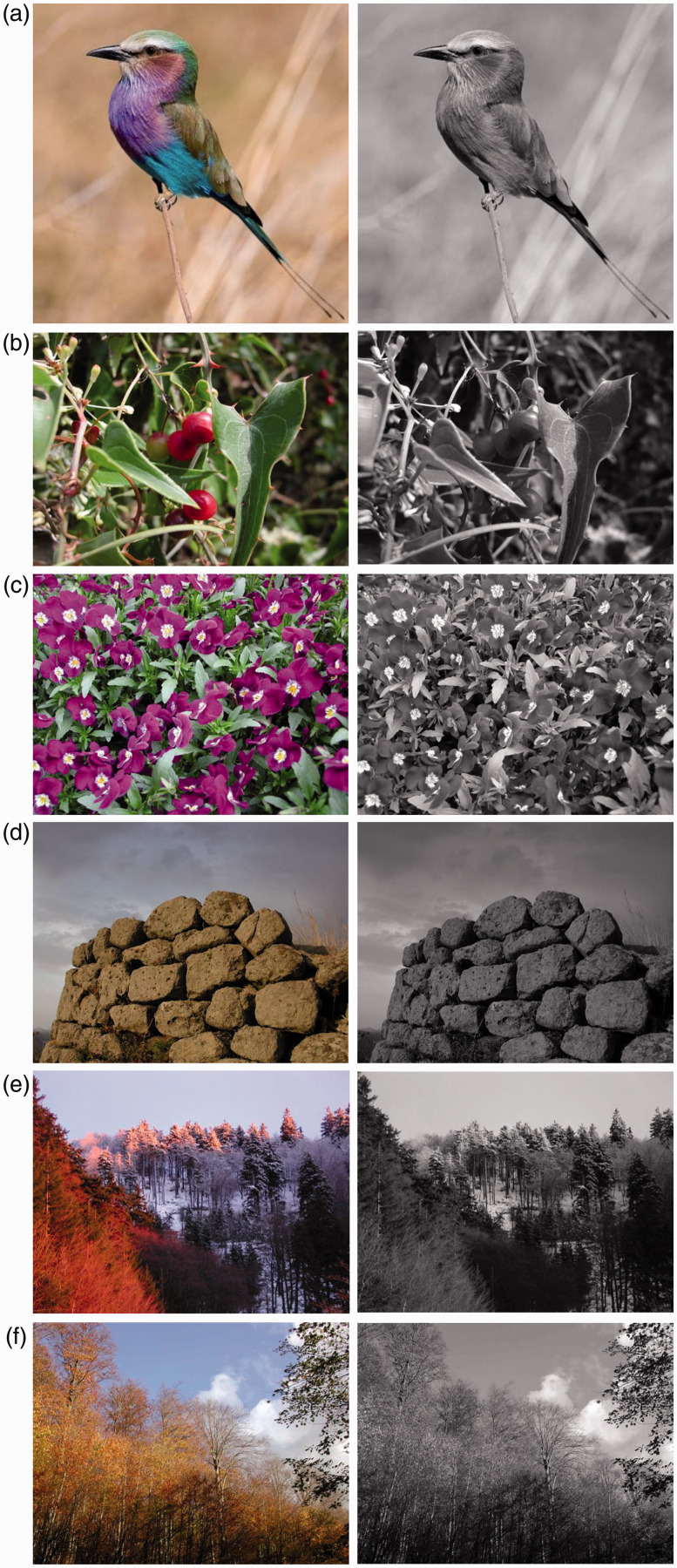
Colors help to segment, locate and organize living organisms into parts and into a multiplicity of fragments. (a) Moreover, the use of color makes the foraging for food easier; (b) contributes to enhance the figurality, the illumination and the 3D appearance; (c) makes objects appear illuminated and brighter; (d) defines the illumination, the light reflexes, the time of the day, the weather, the season, making complex scenes like those of autumn ((e) to (f)) more vivid.

What would our world look like without color? Surely, a world deprived of color would be missing of some of the very crucial elements of life. Not only do colors allow us to see the world more precisely, but also they enhance emergent qualities that would not exist in a world without them (Pinna, 2006; Werner, Pinna, & Spillmann, 2007). This can be easily seen in Figure 1(a), where different parts and phenomenal attributes of objects emerge by means of color variations.

In Figure 1(b), not only does color increase the figural salience of the fruits within the surrounding leaves and vine-branches, but also it indicates which one of them is ripe enough to be eaten. Color variations allow a depth segregation of the components.

Again, the salience and depth segregation of the flowers presented in Figure 1(c) is strongly enhanced if compared to their achromatic counterparts. Color also promotes the emergence of the illumination and of the light reflected by flowers. Both of these phenomenal properties in synergy contribute to increasing the volumetric 3D appearance and the figurality of the flowers that can be seen and distinguished at a long distance.

The use of color clearly helps to define the effect of illumination as observable in Figure 1(d). Color also enhances the chromatic properties of the light reflected by the Nuraghe (Sardinian monument: Paleolithic tower-like building) and, consequently, it emphasizes the effect of the time of the day (late afternoon in this picture). What is worthwhile noting is that the chromatic Nuraghe appears much brighter and lighter than the cloudy sky. These qualities are lost in the dark achromatic version of the Nuraghe. Indeed, color also enhances the 3D appearance.

The illumination and its properties, determined by colors, are even more clearly perceived in Figure 1(e). Here, the achromatic counterpart does not reveal any illumination, at least not in terms of the richness of properties perceived in Figure 1(d). The color of the reflected light not only does it suggest the time of day but also it indicates the latitude of the region.

By employing color, it is also possible to make visible other complex and indirect scene information like that caused by varying seasons. In Figure 1(f), autumn is easily recognized due to the color of the leaves. This information is not observable in the black-and-white picture.

In the animal kingdom, the ability to perceive colors, not just luminance differences, has evolved independently several times over millions of years (Nathans, Thomas, & Hogness, 1986) underlining how color vision has some fundamental and specialized functions, which provide biological advantages. But what are these advantages? What is color good for? To understand these general questions (cf. Chalupa & Werner, 2003; Dresp & Langley 2006, 2009, 2010), some more specific questions need to be asked (Regan et al., 2001): How is color related to segregation between objects and their backgrounds and to discrimination among objects and their parts? Related questions like these ones are of crucial importance for adaptive visual processing and understanding of the real world.

Indeed, the great variety of colors in animals and plants highly suggests that coloration may play a very relevant and crucial role for their survival (De Valois, 2004; Endler, 1991; Poulton, 1890). Indeed, coloration is a cue for species identification, sexual dimorphism and sexual attraction within the same species. Coloration is also fundamental in highlighting the presence of other individuals, in signaling that they are dangerous, poisonous or unpalatable, and they are also useful to show the strength and age of potential contenders. However, in nature, there is often the case where animals instead of advertising their presence with warning colors, they use colors for cryptic camouflage as a defense against predation. In fact, in the course of millennia, they have evolved a very complex set of unobtrusive colors and patterns that allow them to blend into the environment.

In short, color perception manifests high biological adaptive fitness and capability of an individual of a certain genotype to reproduce and propagate its genes to the next generation. To achieve these goals, the color system uses reflectance information in order to code the presence, the position and the figural properties of an individual. Moreover, it facilitates the ability to segregate, locate and organize the world into objects and parts. Pinna and Reeves (2013) suggested that the more general biological questions about color could be reduced to an epistemologically lower level underlying the biological role of color in perceptual organization. According to this level, new implicit and primitive questions arise: What are the factors underlying the visual organization induced by color that determines and favors evolutionary and adaptive functions? What is the basic perceptual structure and its components, on whose foundation the main evolutionary functions of color occur?

### The Problem of Perceptual Organization and the Role of Color in Text Reading

The problem of perceptual organization is traditionally related to the complexity of the following question, first suggested by Wertheimer (1912a, 1912b, 1923): Why do we perceive a world made of objects such as people, cities, houses and trees, and not composed of scattered differences of luminance, colors, edges and bars? The answer to this question is based on the well-known ‘principles of grouping’ – proximity, similarity, good continuation, closure, convexity, exhaustiveness, symmetry, Prägnanz and past experience – resulted from Wertheimer’s (1922, 1923) studies, whereas other fundamental principles of figure-ground organization – surroundedness, size, orientation, contrast, symmetry, convexity, and parallelism – were discovered by Rubin (1921).

**Figure 2.**
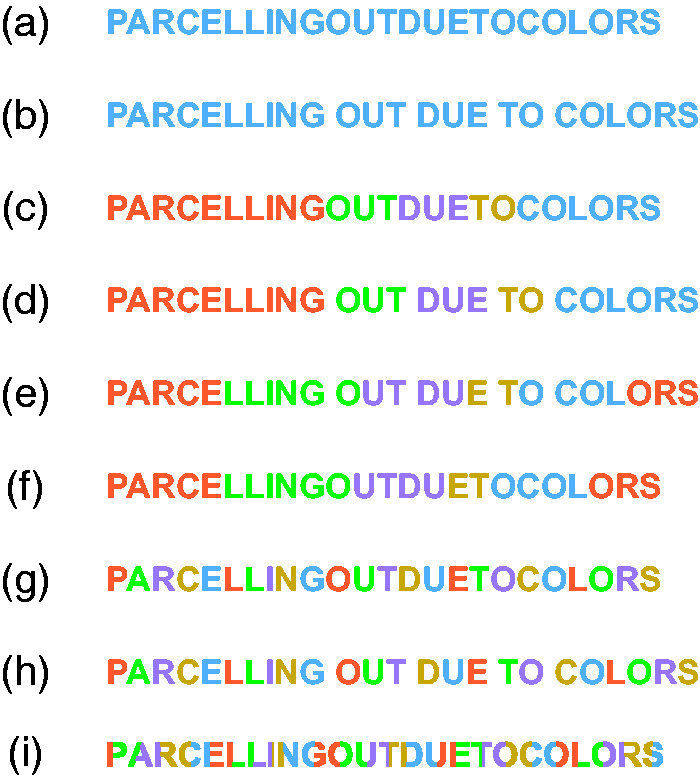
Examples showing the role of color by chromatic similarity in the reading process.

The role of color in Gestalt grouping was usually considered as one among the many attributes involved in triggering the similarity principle, although grouping by color is believed to be less effective if compared with other attributes such as shape, luminance or motion (Arnheim, 1997; Helson, 1947; Katz & Revesz, 1921; Koffka, 1935; Vicario, 1968). In a recent study, Pinna and Deiana (2014) investigated the fundamental role of color in visual organization and segmentation, by imparting spatial rhythm and unification, in grouping and ungrouping wholenesses, in uncrowding shapes, in making implicit shapes explicit as a whole, in shaping and reshaping the same pattern of stimuli in different ways. These findings were strengthened and extended also by studying the way color affects the reading process. More particularly, in this earlier study, we explored visual grouping and wholeness in relation to the similarity principle under the conditions illustrated in Figure 2.

Figure 2(a) shows a long monochromatic set of letters where no blank spaces separate words. This is a condition which is hard to read. However, by introducing a blank space as in Figure 2(b), the reading becomes easier due to the proximity principle of grouping that promotes a sort of visual rhythm thereby facilitating the reading process. In Figure 2(c) again words are not separated by any blank space; however, all words are colored in different colors, so since the long sequence is now segmented, a new rhythm is given by chromatic similarity in such a way that reading becomes easier, even easier than for the text proposed in Figure 2(b). In Figure 2(d), words are still colored by different colors and in addition, each word is also separated by the blank space so that this is now the easiest reading condition among the other conditions. On the other hand, to make a text difficult and even more difficult to read, it is sufficient to pit one principle against the other as in Figures 2(e) to (i). For example, in Figure 2(e), the text is made unreadable or very difficult to read because of the chromatic similarity which splits the words into two colors and strategically makes the terminating color of each word the initial color of the subsequent one. This, of course, generates within the set of words a segmentation and a visual rhythm, which can be disturbing and confusing to read. To make the reading process even more difficult, in Figure 2(f), the same chromatic condition as in Figure 2(e) is maintained but, this time, no blank space is left between words so that, here the chromatic similarity enhances the emerging of non-words and tends to camouflage words that instead are there. Three control stimuli are also presented in Figures 2(g) to (i). In these controls, the chromatic dissimilarity among adjacent letters fragments each word. In fact, these control conditions tend to make reading difficult. As a result, the text in Figures 2(g) to (i) is more difficult to read than the text presented in Figure 2(a) and the text in Figure 2(h) is more difficult to read than that presented in Figure 2(b).

On the basis of these conditions, Pinna and Deiana (2014) studied how color influences reading time, reading easiness and reading comprehension when chromatic similarity is pitted in favor or against other principles of Gestalt grouping. The principles taken into account were proximity (distance between two adjacent words and breaking off each word by adding a blank space), element connectedness (underline typeface) and past experience. However, in addition, other similarity attributes were used in comparison like orientation (italic font) and width (bold font). Their results demonstrated that chromatic similarity can affect the process of segmentation of words and, above all, phenomenal grouping and shape formation. Furthermore, it has been proved that color wins against all of the known typesetting similarities such as underline, italic and bold fonts playing together. This means that color not only is one among the many factors of grouping but it is also an essential element useful for the foundation of a more complex organization whose aim is to create wholeness, parts-whole formation and segmentation.

Based on the previous results, the main purpose of this study is to extend this experimental protocol to subjects affected by developmental dyslexia, a neurological disorder that specifically impedes the development of expertise in reading skills (Gabrieli, 2009; Lyon, Shaywitz, & Shaywitz, 2003). Dyslexia is a reading disability thought to be related to a defect in the brain’s ability to process graphic symbols. In short, it is a reading and learning disability that modifies the way the brain processes written material notwithstanding normal intelligence, adequate socio-cultural conditions and preserved elementary sensory skills (American Psychiatric Association, 2013; World Health Organization, 1993). It often co-occurs with phonological deficits (Snowling, 2001) that persist in adult life (Paulesu et al., 2001; Ramus et al., 2003). Although it is fairly clear which classes of phonological tasks are more sensitive in bringing about a deficient performance in dyslexics (cf. Pennington, Van Orden, Smith, Green, & Haith, 1990), the peculiar nature of cognitive deficits underlying these faulty performances remains to be established (see also Frith, 1999).

Color has been used in reading tasks with dyslexic subjects in a number of different ways (Irlen, 2010; Jansky, 1958; Wilkins, 2003) and for different purposes (Irlen, 2010; Kruk, Sumbler, & Willows, 2008; Scott et al., 2002) either to facilitate normal readers performance and for improving reading and increasing speed and fluency in persons with visual stress and dyslexia. The so-called visual stress syndrome (Irlen, 1991; Singleton & Henderson, 2007; Singleton & Trotter, 2005) is often observed in dyslexic subjects, although this concept remains controversial (see Uccula, Enna, & Mulatti, 2014). This idea has led to a widespread use of colored filters to alleviate and ameliorate reading disorders. It is in fact suggested (Hoyt, 1990) that symptoms of the visual stress syndrome are provoked by sensitivity to certain frequencies of the light spectrum.

The practical applications of colored filters for reading have led to the use of lenses and overlays. The former approach uses lenses tinted with color placed on eyeglasses worn while reading. Colored overlays are plastic reading sheets tinted with color and placed over a text (Wilkins, 2003) to filter the light. It has been reported by many dyslexic subjects that colored overlays can help in a wide range of difficulties arising when reading (Dickinson, Gregor, & Newell, 2002; Gregor & Newell, 2000; Jeanes et al., 1997; Kriss & Evans, 2005; Wilkins, 2002; Wilkins et al., 1994; Wyman, 2013).

Besides, Gregor and Newell (2000) and later also Dickinson et al. (2002) stressed the importance of changes related to perceptual organization in the presentation of a text. They highlighted how visual changes are essential to alleviate some of the problems caused by dyslexia. On the basis of these claims, other studies are especially focused on specific designs for computer screen texts where brightness and color are used in combination with other parameters, such as font type, character, line and paragraph spacing, column width and language content. All these parameters, which have been proved to be effective for dyslexics, have been adjusted in such a way to make web texts more accessible to dyslexics without affecting other users (Rello, 2013; Rello & Baeza-Yates, 2015). For example, it has been stated that larger texts and larger character spacing help dyslexics and normal readers to read faster (Rello & Baeza-Yates, 2015). “The Web Content Accessibility Guidelines 1.0 (WCAG)” determined the correct parameters and algorithms for brightness contrast and color difference for a better readability of a text. However, Rello and Baeza-Yates (2015) stated that these algorithms may have detrimental effects on dyslexics. Due to color contrast, in fact, many dyslexics experience discomfort while reading, as also reported in visual stress syndrome.

Although these studies are related to color and perceptual organization, they do not investigate directly and specifically the role of color in terms of the three basic aspects: wholeness, parts-whole formation and segmentation. None of them explored how dyslexic readers perform under conditions like those illustrated in Figure 2. In the next sections, the role of color in reading and comprehension tasks has been investigated in normal and dyslexic children and adults.

## Methods

### Subjects

Four groups of naive subjects took part in this study: (a) 50 dyslexic children (27 boys and 23 girls, mean age 10.2 ± 4.5 years), (b) 50 dyslexic adults (22 boys and 28 girls, mean age 25.4 ± 4.2 years), (c) 50 children skilled at reading (24 boys and 26 girls, mean age 11.2 ± 5.5 years) and (d) 50 adults skilled at reading (25 boys and 25 girls, mean age 25.9 ± 7.6 years).

Subjects of the first two groups manifest the following symptoms (American Psychiatric Association, 2013). (a) Inaccurate or slow and effortful word reading characterized by reading single words aloud incorrectly, or slowly and hesitantly, frequently guessing words when reading, having difficulty in sounding out words. (b) Difficulty in understanding the meaning of what is read. For example, not understanding the sequence, relationships and deeper meaning of the text even if reading accurately. (c) Difficulty in spelling. For example, they may omit, add or substitute vowels or consonants. (d) Difficulties with written texts, making multiple grammatical or punctuation errors within sentences, or employing poor paragraph organization and having a lack of clarity in written expression when reporting their ideas.

All dyslexic participants had previously undergone a complete neuropsychological assessment to establish the diagnosis of developmental dyslexia. All children had been diagnosed as dyslexic based on standard inclusion and exclusion criteria (World Health Organization, 1993). Their performance in reading was two (or more) standard deviations below the mean in at least one of the age-standardized Italian reading tests included in the battery (word, non-word and text reading), and their non-verbal or performance IQ was above 85. Performance IQ was estimated by the Italian adaptation of the Wechsler Intelligence Scale for Children-revised (WISC-R; Wechsler, 1994), or Cattell’s “Culture Free” test (Cattell, 1979). Comorbidity with ADHD or other psychopathological conditions was excluded, based on standard diagnostic criteria (American Psychiatric Association, 2013).

All participants, dyslexic readers and normal efficient readers, were classified by mean of The Dyslexia Screener (TDS), a rapid 5-minute test, which helped to fit children and adults into categories of either non-dyslexia, different degrees of dyslexia, specifying the three major subtypes: dysphonetic (subjects with problems in word-attack coding, thus, being more ‘auditory’), dyseidetic (subjects with problems with whole-word coding, thus, being more ‘visual’), or mixed (subjects being both dyseidetic and dysphonetic), as specified by Boder (1971). The TDS, although having a very high predictive power (87%) for identifying poor readers who are dyslexic, has also been validated using the Woodcock-Johnson standardized reading tests. Children were classified as dyslexic in grades. Some were dysphonetic, others were found dyseidetic, but some others were mixed, being both dysphonetic and dyseidetic, ranging in severity from borderline or markedly below normal. All dyslexics, both children and adults, were at an appropriate grade for decoding reading. Reading efficiency was assessed by the MT Reading test (Cornoldi & Colpo, 1995).

All subjects in each group had normal visual acuity or vision corrected with lenses because of slight myopia and astigmatism. All had received an ophthalmic, optometric and orthoptic assessment in order to spot any significant non-corrected visual problem. They also had normal color vision tested on the Ishihara 24 test plates and on the Farnsworth-Munsell 100 hue Color Vision Test. In order to participate to this study, they also had to have normal intelligence as verified by standardized tests administered by the school, and not known visual abnormalities, or obvious behavioral or neurological disorders of any kind.

This research has been approved in each school from which subjects were recruited following an approval documentation, which stated that this research was not meant to test personality nor emotion nor the intelligence nor familiar social issues, and it was stressed that it was only meant to test the effects of chromatic accentuation in different categories of readers.

We recruited children and adults dyslexics thanks to schools, teachers, therapists, headmasters and under previous informed consent signed by all participants and by parents or legal guardian in case of children in compliance with the Helsinki declaration. Although they were all certified as dyslexics, they were found to have different subtypes of dyslexia. In other terms, some of them were pure dyslexics, some were dysphonetic and dyseidetic. In addition, among them, after years of school education, there were some subjects who were adequately compensated in such a way that they had learned how to deal with their deficiency to overcome their problems efficiently enough so as to give a fairly adequate performance. On the other hand, some others were surely not so efficiently compensated or at least they were less compensated so that they had more problems in overcoming the reading difficulties. Among these subjects, some were also disorthographics or dyscalculics. However, all subjects were allocated in the right group according to their main characteristics: being a child/adult dyslexic or being a child/adult normal efficient reader. Criteria for allocation came from further sources beyond those previously described. First of all, all the details learned directly from reports given by subjects themselves or reported by school teachers and parents together, plus their specific medical documentation, helped to allocate subjects.

### Stimuli

The stimuli were created using five different equiluminant colors (∼72.27 cd m^2^): brown (CIE chromaticity coordinates *x*,*y* = 0.49, 0.40), blue (0.17, 0.19), green (0.34, 0.51), purple (0.35, 0.24) and red (0.55, 0.37). For each observer, we determined the luminance match for the two contours with the luminance background to be tested using a variation of the minimally distinct border technique of Boynton and Kaiser (1968). The overall sizes of the figures were each ∼5 deg. The luminance of the white background was ∼122.3 cd m^2^. Black contours had a luminance value of ∼2.6 cd m^2^. Stimuli were displayed on a 33-cm color CRT monitor (GDM-F520 1600 × 1200 pixels, refresh rate 100 Hz: Sony Corporation, Tokyo, Japan), driven by a MacBook Pro computer (Apple Ins., Cupertino, CA, USA) with a GeForce 8600M GT (Nvidia Corp. Santa Clare, CA, USA). The monitor was calibrated using a CS 100 Chroma Meter colorimeter (Konica Minolta, Tokyo, Japan) and procedures set out in Brainard, Pelli, and Robson (2002).

The stimuli were presented in random order, as described in the Procedure section, on a computer screen with ambient illumination from a Daylight fluorescent light (250 lux, 5600°K: Osram, Munich, Germany).

As in Pinna and Deiana (2014), the basic stimulus text was the well-known ‘the War of Ghosts’ (see below for the full text in English) by Bartlett (1932) translated in Italian.

One night two young men from Egulac went down to the river to hunt seals and while they were there it became foggy and calm. Then they heard war-cries, and they thought: “Maybe this is a war-party.” They escaped to the shore and hid behind a log. Now canoes came up, and they heard the noise of paddles and saw one canoe coming up to them. There were five men in the canoe, and they said:

“What do you think? We wish to take you along. We are going up the river to make war on the people.”

One of the young men said, “I have no arrows.”

“Arrows are in the canoe,” they said.

“I will not go along. I might be killed. My relatives do not know where I have gone. But you,” he said, turning to the other, “may go with them.”

So one of the young men went, but the other returned home.

And the warriors went on up the river to a town on the other side of Kalama. The people came down to the water and they began to fight, and many were killed. But presently the young man heard one of the warriors say, “Quick, let us go home: that Indian has been hit.” Now he thought: “Oh, they are ghosts.” He did not feel sick, but they said he had been shot.

So the canoes went back to Egulac and the young man went ashore to his house and made a fire. And he told everybody and said: “Behold I accompanied the ghosts, and we went to fight. Many of our fellows were killed, and many of those who attacked us were killed. They said I was hit, and I did not feel sick.”

He told it all, and then he became quiet. When the sun rose he fell down. Something black came out of his mouth. His face became contorted. The people jumped up and cried.

He was dead.

**Figure 3.**
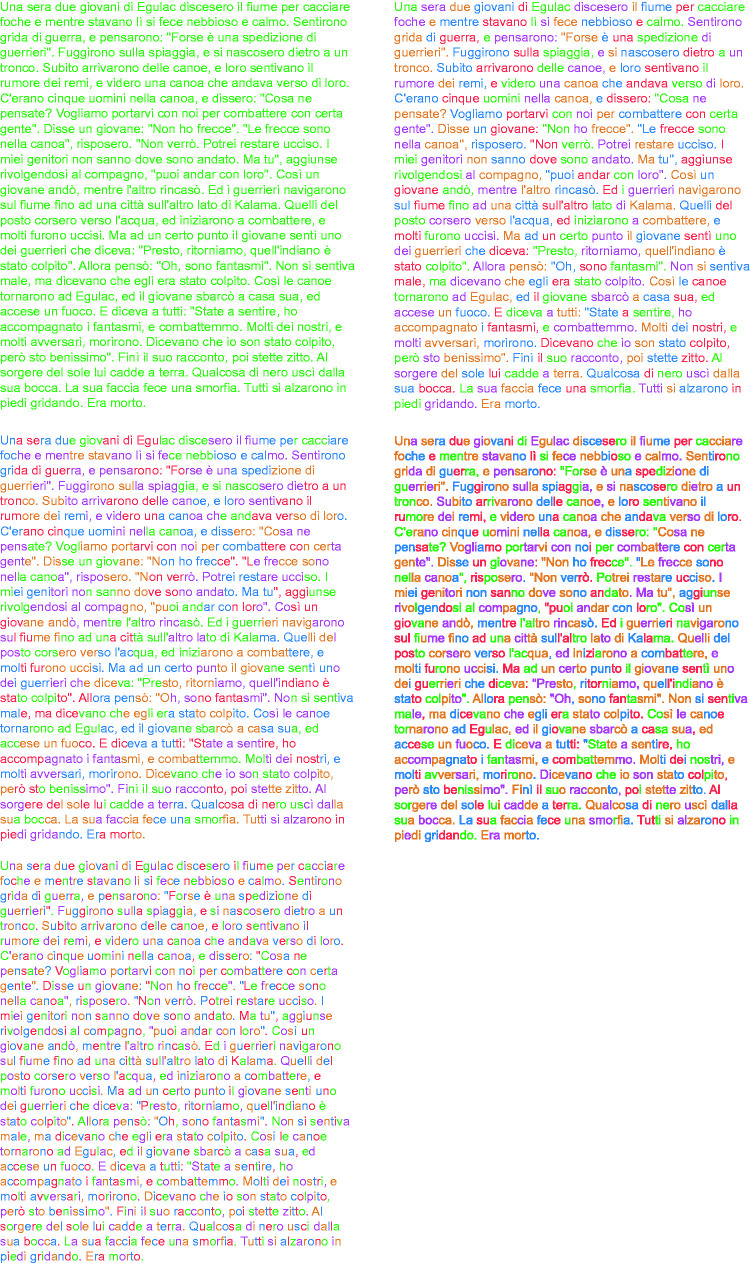
The five ((a) to (e)) chromatic conditions used as stimuli respectively for the word (left) and non-word (right) conditions.

The stimulus text was varied as follows (see Figure 3): (a) word condition, that is, words separated by a blank interspace, and (b) non-word condition, that is, each non-word was obtained by mixing and reversing randomly adjacent letters within each word of The War of Ghosts (not illustrated). The non-word condition was not studied in Pinna and Deiana (2014).

Colors were used to create five chromatic conditions as follows (Figure 3): (a) monochromatic condition – the whole text is colored with only one of the five equiluminant colors (green) previously described (Figure 3(a)); (b) word condition – each word in the text is colored in different color (Figure 3(b)); (c) half word condition – half word is colored with a color different from the one of the second half whose color is the same of the one of the first half of the next half word (Figure 3(c)); (d) syllable condition – every syllable has its own color (Figure 3(d)); (e) letter condition – each letter is of a different color from the adjacent ones. Differently, from a previous work (Pinna & Deiana, 2014), the syllable condition is now included with the purpose to explore possible effects in the four groups of participants

Through these five conditions, color was used to pit chromatic similarity in favor (cf. the word condition with respect to the monochromatic control) or against the grouping principles of proximity (the distance between two adjacent words or the breaking off of the words by adding a blank space), of the element of connectedness (Palmer, 1999) and of past experience. As a matter of fact, the half word condition uses the color to split (ungroup what is grouped by proximity) the word into two parts, each of them are grouped by chromatic similarity respectively to the previous and the next word. Also, the syllable and the letter conditions play with chromatic similarly but less strongly. Within these last conditions, the ungrouping effect is strong; nevertheless, it is not reinforced by the grouping by similarity with the previous and next word as it happens in the half word condition.

To sum up, the five chromatic variations of the stimulus text were aimed to create wholes (monochromatic and word conditions), parts-whole organizations (word condition) and fragments (syllable and letter conditions) and as such they were expected to influence the reading process in time, number of errors and comprehension of the text.

### Procedure

Subjects’ tasks were (a) to read the text as clearly, accurately and as quickly as possible in a loud voice (reading task) and (b) to answer a multiple-choice comprehension test (comprehension task). The dependent variable used was reading time, number of reading errors (phonetic, omissions, etc.) and number of incorrect answers to the comprehension task on a scale from 0 to 10, namely, the higher the score, the higher the number of incorrect answers.

All adult subjects were tested in the laboratory, while all children were tested in their own school, in a silent room and under the same conditions set up in the lab. Each subject was given two stimuli to read: a word and a non-word stimulus. This entails that 10 subjects of each group were involved in each couple of stimuli, for a total of 50 subjects involved in the five couples of word and non-word conditions. The presentation order of the two stimuli composing the couple (i.e., which stimulus, word or non-word, was presented as a first or as a second) was randomized and their presentation was separated by an hour delay to let the subject to rest after the first reading and comprehension tasks. Although some kind of visual contamination in between the two stimuli might have occurred, it is assumed to be very small and counterbalanced by the random presentation. Moreover, the two stimuli (word and non-word) were never presented under the same chromatic conditions to avoid undesirable chromatic contaminations.

All conditions were displayed in a fronto-parallel plane at a distance of 50 cm from the observer. There was no time limit.

## Results

The main findings of this work are shown by describing separately the main outcomes of each dependent variable (reading time, reading errors and number of incorrect answers in the comprehension test) for the five chromatic conditions and for the four groups of subjects (normal children and adults and dyslexic children and adults).

### Reading Time

**Figure 4.**
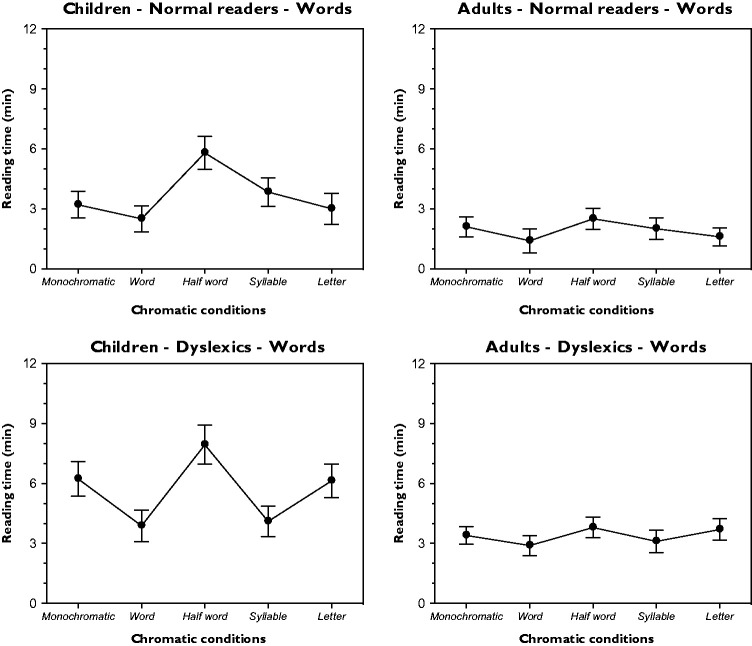
Results for the reading time under the word condition as a function of different chromatic variations and for different groups (children and adults, normal and dyslexic readers).

The outcomes of the reading time under the word condition as a function of different chromatic variations and for different groups (children and adults, normal and dyslexic readers) are illustrated in Figure 4. They clearly support previous results (Pinna & Deiana, 2014) according to which the similarity and dissimilarity attributes and, thus, the grouping/ungrouping dynamics imparted by color in the different conditions strongly affect the time needed to read the War of Ghosts. A one-way analysis of variance (ANOVA) for each group demonstrated significant variations within the five chromatic conditions – children-normal readers: *F*(4, 36) = 17.7, *p* < .001; adults-normal readers: *F*(4, 36) = 13.2, *p* < .001; children-dyslexics: *F*(4, 36) = 15.9, *p* < .001; and adults-dyslexics: *F*(4, 36) = 13.2, *p* < .001. The Fisher PLSD post hoc analyses of the possible pairs were significant (*p* < .05).

By considering the monochromatic condition as a control, both normal and dyslexics readers, either children and adults, read the word condition (i.e., when each word is popped out through different colors) faster than the control. On the contrary, the reading time increases in the half word condition. These results were expected. In fact, the same chromatic similarity/dissimilarity principle operates in opposite directions, that is, by grouping or by splitting and ungrouping each word, thus making the reading process faster or slower than the control. The effect of chromatic similarity/dissimilarity is annulled in the letter condition, since each letter, having a different color from the adjacent one, is on its own. The result is a visual structure in some way very similar to the monochromatic condition, where the color of each letter does not change. In fact, since each component of the letter condition has a different color, all the letters are similar in their dissimilarity. As a consequence, the chromatic grouping/ungrouping push/pull effect strong, which is strong in the word and half word, is now reduced or totally annulled, thus creating a homogeneous heterogeneity isomorphic to the monochromatic control.

Finally, the syllable condition appears to be as something special and somewhere in between word, half word and letters. To better understand and predict the results, it can be useful to clarify what is a syllable. This remark is also useful to understand our choice to include the syllable variation in our experiments. A syllable is a unit of organization for a sequence of speech sounds. This means that a syllable is a perceptual unity, something distinct, namely, the result of phonological grouping. This low-level grouping unit represents a sort of building primitive unit for each word. This is in some way similar to the grouping effect due to colors in the previous conditions that can be considered as building blocks of colors, which combined in different ways, speed up or slow down the reading time.

As such, the syllable can influence the rhythm of the spoken language, its prosody but also its poetic meter and its stress arrangement. The spelling of syllables, as primitive units, during the study of reading is of crucial importance for all children and, all the more reason, for dyslexics. Moreover, it is also true that the spelling of syllable becomes less and less essential during the learning progress, but not for dyslexics (see Jiménez-Fernández, Gutiérrez-Palma & Defiora, 2015). This is clearly shown in our results where the reading time of the syllable condition is in both dyslexics children and adults faster than the control and the letter condition and comparable in speed with the word condition. While, by contrast, it is slower for normal children and adults. This is a major difference between normal readers and dyslexics. A further, although expected difference, is related to the absolute values of the reading time, which is significantly slower in dyslexics children and adults.

In summary, the results of the reading time demonstrate that color can strongly affect wholeness and segmentation during the reading process according to a scale where the highest and lowest poles belong respectively to the word and half word conditions. Finally, our results demonstrate that color can be a significant tool useful to improve the reading performances of both normal and dyslexic readers.

**Figure 5.**
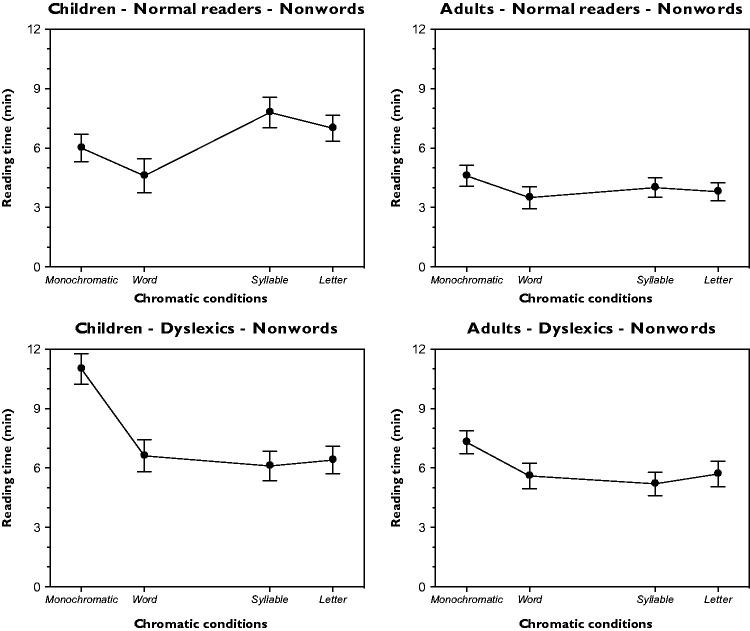
Results for the non-word reading time as a function of different chromatic conditions and for different groups.

The results of the reading time under non-word conditions are now illustrated in Figure 5. They show again the significant strength of chromatic similarity and dissimilarity in grouping/ungrouping elements. A one-way ANOVA showed significant variations within the five chromatic variations for the non-word condition and for each group – children-normal readers: *F*(3, 27) = 16.5, *p* < .001; adults-normal readers: *F*(3, 27) = 16.2, *p* < .001; children-dyslexics: *F*(3, 27) = 15.9, *p* < .001; and adults-dyslexics: *F*(3, 27) = 14.2, *p* < .001. The Fisher PLSD post hoc analyses of the main pairs were significant (*p* < .05).

The following conditions were studied here: monochromatic, word (although the right term should be “non-word,” we leave “word” to make easy the comparison among conditions and tasks), syllable and letter. The half word variation was not included for obvious reasons, since this variation was aimed to pit the chromatic similarity, belonging to a low perceptual domain, against the high-level cognitive domain of word recognition, considered as related to what Gestalt psychologists called “past experience.” Therefore, the role of color is now focused on a reading process without meanings. Hence, chromatic similarity is now pitted against the grapheme–phoneme conversion rules mainly aimed to read non-words.

A word is usually processed following three main routes (cf. Friedmann, Dotan, & Rahamim, 2010; Friedmann & Haddad-Hanna, 2012; Friedmann & Rahamim, 2007; Kohnen, Nickels, Castles, Friedmann, & McArthur, 2012): (a) lexical route (orthographic input lexicon to phonological output lexicon), (b) lexical-semantic route (orthographic input lexicon to phonological output lexicon across the semantic system), and (c) non-lexical route (grapheme–phoneme conversion). Typically, the lexical and lexical-semantic routes successfully process all words within a reader’s orthographic input lexicon but it cannot process non-words. In contrast, the non-lexical route successfully sounds out non-words and words that follow typical letter to sound rules but fails to provide accurate pronunciation for irregular words.

Our results demonstrate that the chromatic organization can be pitted successfully in favor or against the grapheme–phoneme conversion process. Although this topic lies outside the scope of our work, the results might suggest a new way to explore and understand how the grapheme–phoneme conversion works.

The findings of the normal and dyslexic readers, both children and adults, support the previous ones by showing the same trends among chromatic conditions, although, in the case of dyslexics, the chromatic variations among words, syllables or letters might confuse making the reading harder (cf. in Figure 5 the values of the monochromatic condition with those of the other conditions). Again, the syllable variation reveals the same differences previously described for normal and dyslexic readers.

### Reading Errors

**Figure 6.**
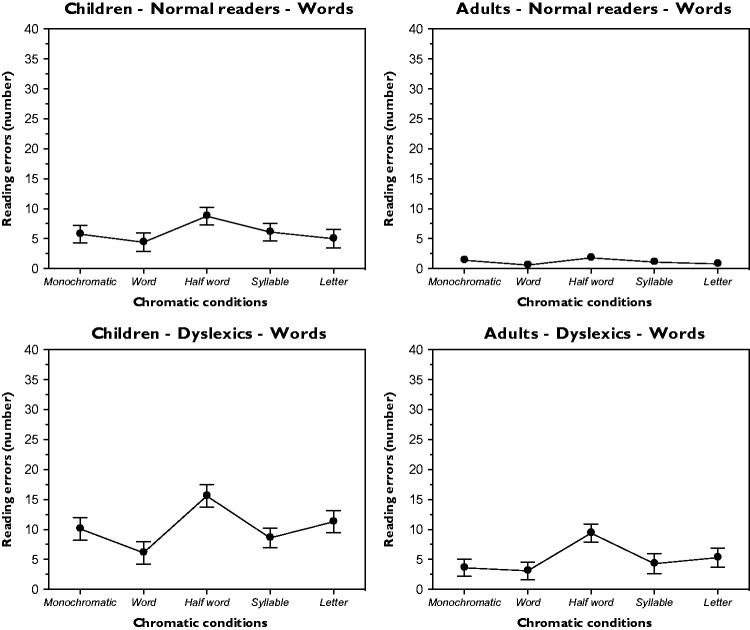
Results for the word reading errors as a function of different chromatic conditions and for different groups.

**Figure 7.**
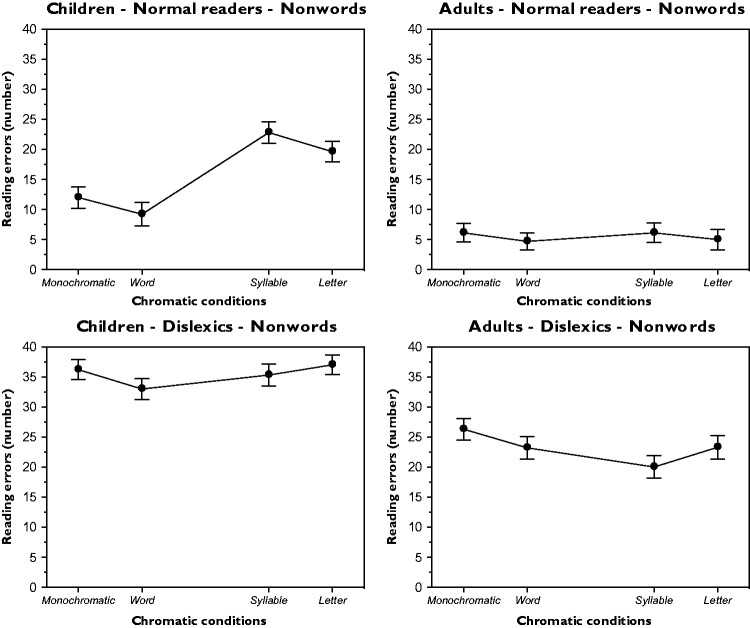
Results for the non-word reading errors as a function of different chromatic conditions and for different groups.

It is spontaneously assumed that the reading time of a text is directly related to its reading difficulties that generate a directly proportional number of reading errors. This entails that the trend of the number of errors is expected to be similar to the trend of the reading time. This is what Figures 6 and 7 respectively report for the word and non-word conditions as a function of the chromatic conditions and for the four groups.

Significant variations within the five chromatic variations were demonstrated through a one-way ANOVA (a) for the word condition and for each group – children-normal readers: *F*(4, 36) = 18.7, *p* < .001; adults-normal readers: *F*(4, 36) = 16.6, *p* < .001; children-dyslexics: *F*(4, 36) = 19.5, *p* < .001; and adults-dyslexics: *F*(4, 36) = 16.8, *p* < .001 and (b) for the non-word condition and for each group – children-normal readers: *F*(3, 27) = 14.5, *p* < .001; adults-normal readers: *F*(3, 27) = 16.2, *p* < .001; children-dyslexics: *F*(3, 27) = 13.2, *p* < .001; and adults-dyslexics: *F*(3, 27) = 16.7, *p* < .001. The Fisher PLSD post hoc analyses of the main pairs were significant (*p* < .05).

Please note the large difference in the absolute number of errors made by normal and dyslexic readers for both word and non-word conditions.

### Comprehension Task (Number of Incorrect Answers)

**Figure 8.**
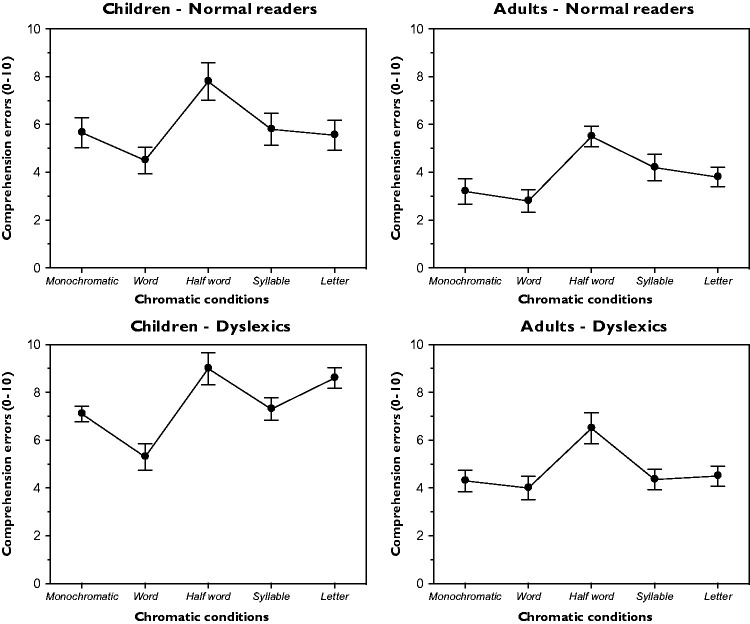
Results for the number of incorrect answers to the comprehension test as a function of different chromatic conditions and for different groups.

Figure 8 illustrates the number of incorrect answers during the comprehension task as a function of the chromatic conditions and the groups of participants. Given the nature of the task, the non-word conditions were absent in this case. A one-way ANOVA for each group demonstrated significant variations within the five chromatic conditions – children-normal readers: *F*(4, 36) = 15.2, *p* < .001; adults-normal readers: *F*(4, 36) = 17.8, *p* < .001; children-dyslexics: *F*(4, 36) = 16.9, *p* < .001; and adults-dyslexics: *F*(4, 36) = 18.8, *p* < .001. The Fisher PLSD post hoc analyses of the possible pairs were significant (*p* < .05).

According to the results of Figure 8 and to the previous findings, the reading time, the number of reading errors and the number of incorrect answers during the comprehension task are demonstrated to be all directly related. In other words, the chromatic condition that requires less time to be read induces also a smaller number of reading errors and a smaller number of incorrect comprehension answers. On the basis of these results, phonological and semantic functions appear strongly related supporting the findings that they are processed in close areas of the bilateral temporal lobes (see Majerus, Poncelet, Elsen, & Van der Linden, 2006; Majerus et al., 2008, 2010).

## Conclusions

In this study, we demonstrated unique and relevant visual properties imparted by chromatic text variations in children and adults either normal or dyslexic readers. It has been shown how the role of color turned out to be crucial in visual organization and segmentation of written text, by influencing reading time, errors and comprehension. This was obtained by pitting chromatic similarity in favor or against the Gestalt principles of grouping involved in a written text (e.g., proximity that occurs after breaking off each word by adding a blank space or past experience that can be varied through word and non-word conditions).

Moreover, it was demonstrated that color can induce wholeness, parts-whole organization and phenomenal fragmentation in words and non-words through several conditions: monochromatic (the whole text colored with only one color), word (each word colored in different color), half word (half word colored with a color different from the one of the second half), syllable (every syllable colored with a different color); and letter (each letter with a different color). The dependent variable considered were reading time, reading errors and incorrect answers to a comprehension test and the results demonstrated that they are all directly related and strongly influenced by the five chromatic conditions. These outcomes were obtained with similar trends by measuring the aleatory variables in four groups of readers: children and adults that could be normal or dyslexic readers. However, a difference between groups of normals and dyslexics emerged in reading words and non-words composed of syllables with different colors that were more effective for the dyslexic groups.

All these results are consistent with previous ones (Pinna & Deiana, 2014; Pinna, Uccula, & Tanca, 2010) showing how color can affect reading in conditions different from the one here presented. The data that emerged from these studies taken together have possible empirical implications. The facilitatory effect of color in reading and in spelling words and non-words could be used to enrich standard teaching methods and rehabilitation strategies in the case of learning disabilities and, more particularly, in dyslexia but also in normal readers. In the present study, we found reliable effects although other studies in larger populations are needed to confirm the present data and to extend them to different conditions where color is used in new ways to generate wholeness, parts-whole organization and fragmentation.

**Figure 9.**
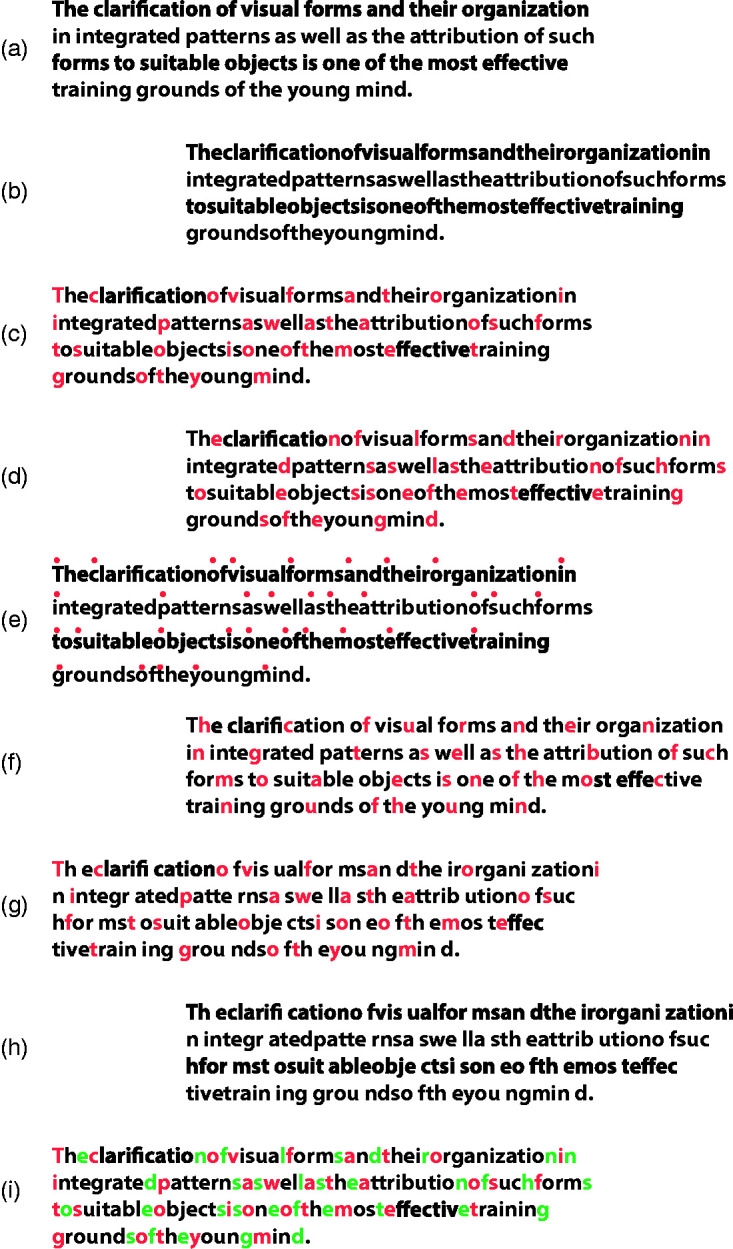
Different kinds of chromatic accentuation influencing the reading process.

A further possible way to investigate chromatic organization in reading is to use it according to the accentuation principle (Pinna, 2010a, 2010b, 2012a, 2012b, 2015; Pinna & Sirigu, 2011, 2016; Pinna, Reeves, Koenderink, van Doorn, & Deiana, 2018) that was shown to be more effective when the accent is dissimilar in color (Pinna, Porcheddu, & Deiana, 2016). In Figure 9(a), an Arnheim’s famous quote is reported and, then, changed by removing the blank spaces in between adjacent words (Figure 9(b)). Then, it has been accentuated through several chromatic conditions: by putting in red the first or the last letter of each word (Figure. 9(c) and (d) and by including a small red circle above the first letter of each word (Figure 9(e)). Furthermore, the chromatic accentuation has been pitted against the principles of proximity and past experience making it difficult to be read (Figure 9(f)), or with words broken in the middle by a blank space in order to make it readable again (Figure 9(g) and (h) for a control) or accentuating each word with two different colors, one placed in the first and the other placed in the last letter (Figure 9(i)).

The chromatic accentuation can clearly make it easier or more difficult to read the quote, by masking or unmasking, disrupting or highlighting the text. Now, if the chromatic accentuation is combined with the chromatic organization studied in this work, the role of color is expected to become ever more effective.

Further developments based on these ideas are related to the colored filters and to the lenses and overlays previously mentioned (see Wilkins, 2003) reported by many dyslexic subjects to be helpful in a wide range of difficulties arising when reading. It is useful to measure and compare the effect of colored filters with those here reported when they are pitted in favor or against each other.

Beyond the possible applied research and clinical applications, the main message of this study goes back to the basic questions raised in the Introduction that is related to role of color in vision. We suggest that our results can contribute to answering those questions. They, in fact, suggest that the main role of chromatic organization is to impart a strong grouping effect on elementary components, much stronger than other Gestalt principles, a role that generates the following outcomes: (a) To group similar chromatic components within an object, determining the emergence of the wholeness. (b) To elicit a parts-whole organization, where both components are not pitted one against the other but complemented and reciprocally reinforced within the whole. (c) To accentuate fragments and to hide the whole by favoring the emergence of single components.

In conclusion, this tripartite role is considered essential to understanding the high neural investment involved in the evolution of color systems, but also the fundamental and specialized functions of color in providing biological advantages and high adaptive fitness. Our results contribute to our understanding of the purpose of color for living beings as well as to revealing its adaptive and perceptual functions.
